# Is There a Role for Basophils in Cancer?

**DOI:** 10.3389/fimmu.2020.02103

**Published:** 2020-09-08

**Authors:** Giancarlo Marone, John T. Schroeder, Fabrizio Mattei, Stefania Loffredo, Adriana Rosa Gambardella, Remo Poto, Amato de Paulis, Giovanna Schiavoni, Gilda Varricchi

**Affiliations:** ^1^Section of Hygiene, Department of Public Health, University of Naples Federico II, Naples, Italy; ^2^Azienda Ospedaliera Ospedali dei Colli, Monaldi Hospital Pharmacy, Naples, Italy; ^3^Division of Allergy and Clinical Immunology, Department of Medicine, Johns Hopkins Asthma and Allergy Center, Johns Hopkins University, Baltimore, MD, United States; ^4^Department of Oncology and Molecular Medicine, Istituto Superiore di Sanità, Rome, Italy; ^5^Department of Translational Medical Sciences, University of Naples Federico II, Naples, Italy; ^6^Center for Basic and Clinical Immunology Research (CISI), University of Naples Federico II, Naples, Italy; ^7^WAO Center of Excellence, Naples, Italy; ^8^Institute of Experimental Endocrinology and Oncology “G. Salvatore”, National Research Council (CNR), Naples, Italy

**Keywords:** angiogenesis, angiopoietins, basophil, cancer, cysteinyl leukotrienes, cytokines, vascular endothelial growth factors

## Abstract

Basophils were identified in human peripheral blood by Paul Ehrlich over 140 years ago. Human basophils represent <1% of peripheral blood leukocytes. During the last decades, basophils have been described also in mice, guinea pigs, rabbits, and monkeys. There are many similarities, but also several immunological differences between human and mouse basophils. There are currently several strains of mice with profound constitutive or inducible basophil deficiency useful to prove that these cells have specific roles *in vivo*. However, none of these mice are solely and completely devoid of all basophils. Therefore, the relevance of these findings to humans remains to be established. It has been known for some time that basophils have the propensity to migrate into the site of inflammation. Recent observations indicate that tissue resident basophils contribute to lung development and locally promote M2 polarization of macrophages. Moreover, there is increasing evidence that lung-resident basophils exhibit a specific phenotype, different from circulating basophils. Activated human and mouse basophils synthesize restricted and distinct profiles of cytokines. Human basophils produce several canonical (e.g., VEGFs, angiopoietin 1) and non-canonical (i.e., cysteinyl leukotriene C_4_) angiogenic factors. Activated human and mouse basophils release extracellular DNA traps that may have multiple effects in cancer. Hyperresponsiveness of basophils has been demonstrated in patients with JAK2^V617F^-positive polycythemia vera. Basophils are present in the immune landscape of human lung adenocarcinoma and pancreatic cancer and can promote inflammation-driven skin tumor growth. The few studies conducted thus far using different models of basophil-deficient mice have provided informative results on the roles of these cells in tumorigenesis. Much more remains to be discovered before we unravel the hitherto mysterious roles of basophils in human and experimental cancers.

## Introduction

**Figure 1:**
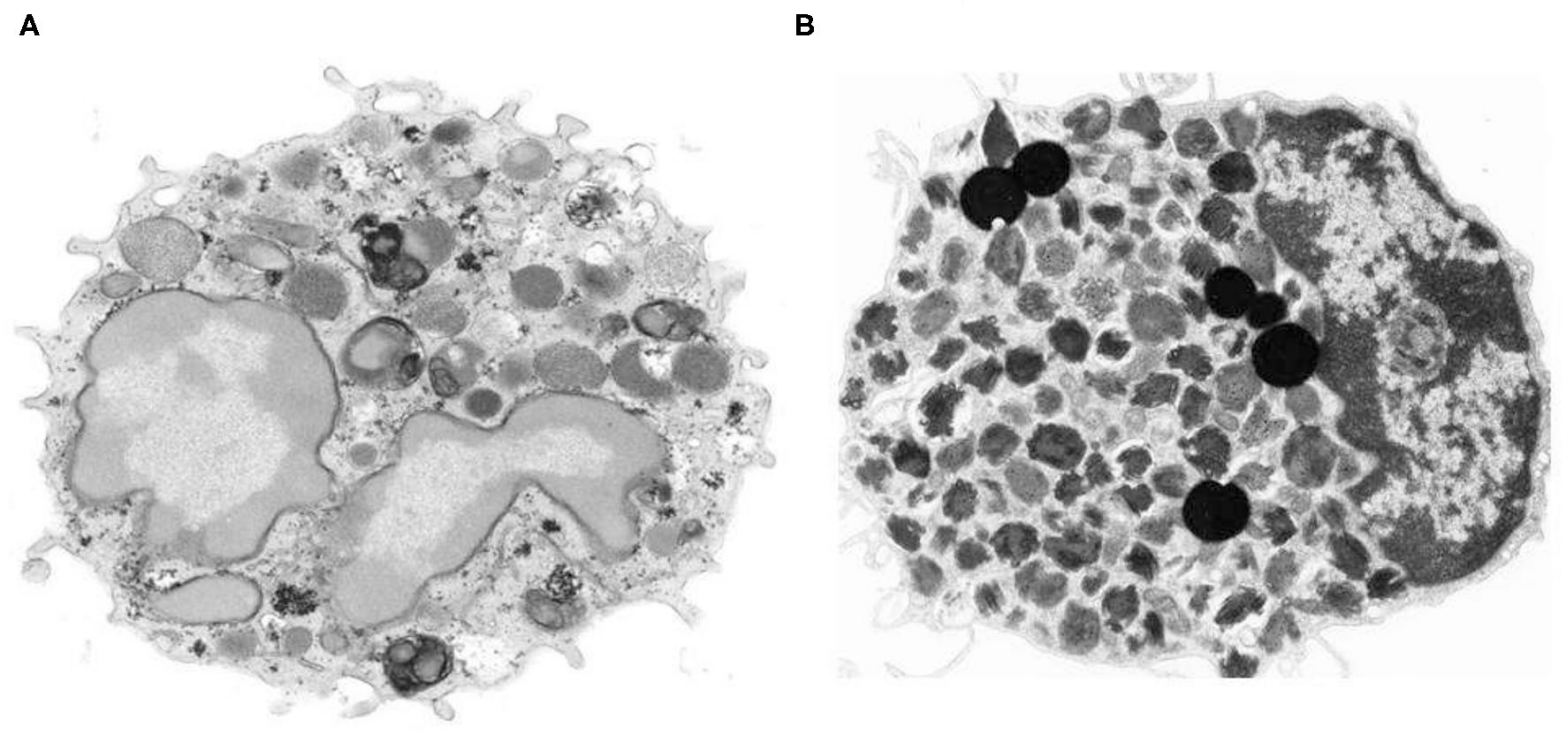
Morphologic and ultrastructural differences between human basophils and mast cells. **(A)** Human peripheral blood basophil shows irregular blunt surface processes and a polylobed nucleus with condensed chromatin pattern. The cytoplasm contains large-membrane bound secretory granules filled with electron dense particles and/or finely granular material (11) X 21,500. **(B)** Isolated human lung mast cell has a narrow surface fold and single lobed nucleus with partially condensed chromatin pattern. The cytoplasm is filled with a large number of membrane-bound secretory granules that have an extremely variable ultrastructural pattern (16, 17). The cytoplasm also contains six non–membrane-bound spherical lipid bodies that are larger than secretory granules, are osmophilic and do not contain scrolls (16, 17) X 14,000. Photos kindly provided by Ann M. Dvorak and reproduced with permission from Marone et al. (18).

Peripheral blood basophils and tissue mast cells were described over 140 years ago by Paul Ehrlich the founder of modern Immunology (1, 2). Basophils have been characterized in humans (3), guinea pigs (4), mice (5, 6), rabbits (7) and monkeys (8). Basophils represent <1% of human peripheral leukocytes, whereas mast cells are ubiquitous in essentially all tissues (9, 10). Basophils share some characteristics with mast cells, including the presence of similar, but distinctive basophilic granules within the cytoplasm (11), surface expression of the full tetramer (α*βγ*_2_) form of the high affinity receptor for IgE (FcεRI) and release of proinflammatory mediators such as histamine and cysteinyl leukotrienes (12, 13). These similarities had initially generated the erroneous hypothesis that basophils represented the circulating precursor/counterpart of tissue mast cells. This concept is no longer accepted, as there is now ample evidence that human basophils and mast cells differ morphologically, ultrastructurally, immunologically, biochemically, and pharmacologically (13–15). In a series of eloquent studies, Ann M. Dvorak carefully described and compared the distinctive morphological and ultrastructural features of human basophils and mast cells (11). Figure 1 illustrates the striking ultrastructural differences between human peripheral blood basophils and lung mast cells (18). In addition to highlighting key ultrastructural differences between basophils and mast cells, Dr. Dvorak also pioneered the characterization of mouse basophils. In fact, there was early belief that questioned the existence of basophils in mice. However, Dr. Dvorak's meticulous work clearly identified mouse basophils as a rare, and often elusive, population of granular cells typically found in bone marrow, with some ultrastructural characteristics similar to human basophils (6, 11, 19).

## Basophil Development

Like other myeloid lineages basophils develop from hematopoietic stem cells in the bone marrow (20). IL-3 is generally viewed as the most important growth factor for basophil development, both in humans and mice (21, 22). Indeed, human and murine basophils can be generated *in vitro by* culturing bone marrow cells in the presence of recombinant IL-3 (23–25). More recently, it has been proposed that thymic stromal lymphopoietin (TSLP) is another growth factor important for the development of mouse basophils (26). Interestingly, IL-3- and TSLP-elicited murine basophils differ in terms of gene expression and functions, suggesting heterogeneity among these basophil populations (27). A study has suggested clinical relevance to this concept in reporting evidence that a small percentage (? 10%) of basophils isolated from asthmatic patients express the TSLP receptor and respond directly to TSLP by releasing histamine and cytokines (28). In contrast, subsequent studies have failed to confirm these findings, showing that human basophils lack expression of the IL-7Rα subunit of TSLP receptor (29) and are unresponsive to *in vitro* stimulation with TSLP (29, 30). Collectively, these findings illustrate some of the controversies yet to be resolved between human and mouse basophils, but also those within each species (13, 31, 32).

## Proinflammatory and Immunoregulatory Mediators/Cytokines Released by Basophils: Human *vs*. Mouse

Many phenotypic markers have been identified on human and mouse basophils, with some minor differences worth noting. For example, basophils from both species express of a variety of activation-linked markers, namely FcεRI (33, 34), but also the degranulation marker, CD63 (35–37), as well as CD203c –an ecto-nucleotide pyrophosphatase/phosphodiesterase (15, 36, 38, 39). In contrast, human basophils express the IgG receptors FcγRIIA, FcγRIIB, and minute amounts of FcγRIIIB, whereas mouse basophils express FcγRIIB and FcγRIIIA (40, 41). As indicated above, both human and mouse basophils express receptors for IL-3 (CD123) (26, 42), but also for GM-CSF (CD116) (43), and IL-33 (ST2) (44–47). Again, it remains unclear whether they similarly express the heterodimeric receptor for TSLP (26, 28–30). To date, only human basophils are reported to express IL-5 receptors (CD125). Human basophils express tropomyosin receptor kinase A (TrkA)(48, 49)—the high affinity receptor for nerve growth factor (NGF) and that this factor mediates functional activity (50). In contrast, there are currently no reports that mouse basophils express TrkA. Both human and mouse basophils share the expression of a variety of chemokine receptors (13, 51–56), but it remains to be determined if mouse basophils express CCR1 and CXCR1 (57). These phenotypic comparisons between human and mouse basophils are summarized in Table 1.

**Table 1:** Comparison of the phenotypic differences between human and mouse basophils^a^^,^ ^b^.

**Phenotypic Marker**	**Human Basophil**	**Mouse Basophil**	**References**
FcεRI	++	++	(34)
FcγRIIA	+	-	(33, 40, 58)
FcγRIIB	+	+	(33, 40, 58)
FcγRIIIA	-	+	(33, 40, 58, 59)
FcγRIIIB	±	-	(33, 40, 58, 60)
CD63	+	+	(35–37)
CD203c	+	+	(15, 36, 38, 39)
(CD123) IL-3Rα	++	++	(26, 42)
(CD116) GM-CSFRα	+	+	(43)
(CD125) IL-5Rα	+	ND	(43)
TSLPR	–	+	(26, 28–30, 32)
(ST2) IL-33R	+	+	(44–47)
CCR1	+	ND	(13, 51)
CCR2	++	+	(13, 51–53)
CCR3	++	±	(13, 51, 61)
CCR5	+	–	(13, 51, 53)
CXCR1	++	ND	(13, 51)
CXCR2	+	+	(13, 51, 62)
CXCR4	+	+	(13, 51, 62, 63)
CRTH2	++	+	(51, 55, 62, 64, 65)
CD200R	+	+	(56, 66)
CD300a	+	+	(67–69)
CD300c	+	+	(68, 70)
CD300f	+	+	(68, 70)
PD-L1	+	ND	(50)
VEGFR2	+	ND	(57)
NRP1/2	+	ND	(57)
TrKA	+	ND	(48, 49)

*ND, not done*.

a *Several key surface markers are used to characterize human [IgE^+^, FcεRI^+^, CCR3^+^, (CD123)IL-3Rα^+^, CD63^+^, CD203c^+^] (15, 36, 57, 61, 71) and murine basophils (FcεRI^+^, KIT^−^, CD49b^+^, CD200R3^+^) (35, 62, 72–76) by flow cytometric analysis*.

b *This table essentially includes the phenotypic characteristics of peripheral blood human and mouse basophils. Phenotypic and/or molecular characteristics of human (50) and mouse basophils in tissues (26, 39, 44, 53, 55, 62) are also included*.

*+: means “expressed”; ++: means “highly expressed”; – means “not expressed”; ±: means “probably expressed under certain circumstances”*.

There are several proinflammatory mediators found preformed in human basophils, including histamine (≃ 1 pg/cell), basogranulin (57, 77) and very low concentrations of tryptase (78). Human (79) and mouse basophils release granzyme B (80), which possesses cytotoxic effects on cancer cells (81, 82). Both human and mouse basophils rapidly synthesize cysteinyl leukotriene C_4_ (LTC_4_) through the 5-lipoxygenase pathway (83). There is evidence that mouse basophils metabolize arachidonic acid through cyclooxygenase activity to form prostaglandin D_2_ (PGD_2_) and prostaglandin E_2_ (PGE_2_) (72, 84). In contrast, there is currently no solid evidence that highly purified human basophils can produce measurable levels of PGD_2_, or any other lipid mediator generated through the cyclooxygenase pathway (85).

With regard to the cytokines secreted by human *vs*. mouse basophils, there are several similarities and differences. First, it is now well-accepted that both human and mouse basophils produce IL-4 (44, 86–97) and IL-13 (44, 89, 92, 94, 97–100). Several reports show that mouse basophils additionally produce IL-6 (44, 73, 101) and TNF-α (44, 73). There are at least two publications reporting TNF-? production by human basophils (88, 102). Numerous attempts to detect this cytokine in supernatants of highly purified human basophils activated by IgE-mediated stimuli have produced negative results. Certainly, other cell types (e.g., monocytes, DCs) produce copious amounts of TNF-? and IL-6 (103, 104), thus making it possible that even low-level contamination with these cells could skew the basophil findings. This issue must be taken into consideration each time any cytokine is reportedly made by basophils. Nevertheless, consistent with the general theme of this review, it is becoming apparent that basophils secrete several angiogenic factors that, when combined with the cytokines thus far mentioned, point to a possible role for these cells in wound healing and/or tumorigenesis (as further discussed below). In particular, vascular endothelial growth factor-A (VEGF-A) (57), angiopoietin-1 (ANGPT1) (105), hepatocyte growth factor (HGF) (44, 106), and amphiregulin (71, 107, 108) are all reportedly produced by human basophils, with some of these also made by mouse basophils (44). Table 2 summarizes the cytokines/factors produced by human *vs*. mouse basophils.

**Table 2:** Comparison of the mediators differently produced by human and mouse basophils.

**Mediator**		**Human Basophil**	**Mouse Basophil**	**References**
Cytokines	IL-3	+	+	(21, 109)
	IL-4	++	+	(44, 87–91, 93–97, 110, 111)
	IL-5	–	ND	(86)
	IL-6	±	+	(44, 73, 89, 95, 101)
	IL-8/CXCL8	+	ND	(86, 88–90, 112)
	IL-13	+	+	(44, 88, 89, 92, 94, 97–100)
	IL-31	+	+	(113)
	TNF-α	±	+	(44, 73, 88, 95, 102)
Chemokines	CCL3	+	+	(100, 114)
	CCL5	+	ND	(112)
	CXCL10	+	ND	(112)
Angiogenic factors	VEGF-A	+	ND	(57)
	VEGF-B	+	ND	(57)
	ANGPT1	+	ND	(105)
	HGF	+	+	(44, 106)
	LTC_4_	+	+	(83, 115)
	Amphiregulin	+	+	(71, 107, 108)
Extracellular DNA Traps		+	+	(116–118)
Granzyme B		+	+	(78, 79)

*ND, not done*.

*+: means “expressed”; ++: means “highly expressed”; – means “not expressed”; ±: means “probably poorly expressed”*.

There are many other fundamentals of basophil biology not discussed herein, but have been extensively reviewed elsewhere (13, 86, 119–123). In this review, we focus our discussion instead on the relatively novel concept of how basophils and their mediators/cytokines may play a role in promoting or limiting tumorigenesis.

## Differences Between Peripheral Blood and Tissue Basophils

The life-span of peripheral blood basophils has been calculated to be relatively short (? 2.5 days in mice) (124) and therefore newly generated basophils are constantly supplied from the bone marrow to the blood (20). It has long been thought that basophils circulate in peripheral blood and are rarely present in tissues unless during specific kinds of inflammation, reported both in mice (62, 73, 124–126) and in humans (50, 127–131). However, this dogma has been recently challenged by a study in mice whereby the authors found that basophils are present in all phases of lung development (44). Lung-resident basophils localize in close proximity of alveoli and, interestingly, exhibit a specific phenotype, highly divergent from peripheral blood basophils. IL-33 and GM-CSF produced in the pulmonary environment mediate the specific gene signature of lung alveolar basophils. Importantly, lung basophils are essential for transcriptional and functional development of alveolar macrophages and their polarization toward the M2 state. The latter finding raises the intriguing possibility that in pathologies characterized by M2 macrophages, as happens in many tumors (132, 133), basophils may be involved in regulating the activity of tumor-associated macrophages. This experimental study has several relevant pathophysiological implications. First, it demonstrates that tissue resident basophils exhibit a specific phenotype, different from circulating basophils. Second, the tissue microenvironment can modulate the specific gene signature of resident basophils through exposure to cytokines (e.g., IL-33, GM-CSF). Third, lung resident basophils can influence the transcriptional and functional development of macrophages. The observations of this elegant study represent important premises for future research.

We would like to suggest that any difference between circulating and tissue basophils should be confirmed in human models, given the differences between human and murine basophils. Moreover, studies are urgently needed to characterize the possible roles of tissue basophils residing in the tumor microenvironment (TME) of different human tumors in order to identify novel potential prognostic biomarkers and therapeutic targets.

## Canonical and Non-Canonical Angiogenic Factors Produced by Basophils

Angiogenesis, the formation of new blood vessels from preexisting ones *via* a process called sprouting, represents one of the hallmarks of cancer (134, 135). Angiogenesis is a highly complex process that may occur under physiological conditions, such as during embryonic development. Pathological angiogenesis can occur in inflammation and in cancer and is driven by the coordinated overexpression of several proangiogenic factors (136). Unlike wound healing, where angiogenesis undergoes a resolution phase, tumor angiogenesis continues abnormally in growing cancers supported by angiogenic factors produced by both cancer cells and infiltrated immune cells (137, 138). The VEGF family (VEGF-A, VEGF-B, VEGF-C, VEGF-D) and their receptors (VEGFR1, VEGFR2, VEGFR3) play intricate roles in initiating and promoting tumor and inflammatory angiogenesis (136). Activated human basophils release substantial amounts of VEGF-A, the most potent proangiogenic molecule (57). VEGFs are potent chemotactic stimuli for human basophils through the engagement of VEGFR2expressed in these cells (57, 139). Thus, VEGFs produced by tumor cells and by several immune cells in TME (136, 139–141) can induce basophil chemotaxis through the activation of VEGFR2 on their surface.

The angiopoietin/Tie receptor system is another player in tumor angiogenesis. Angiopoietins (ANGPTs) are a group of growth factors that are involved in regulating vascular functions (142). ANGPTs and their receptors (Tie1 and Tie2) participate in inflammatory and tumor angiogenesis (143). ANGPT1 binds with high affinity to the Tie2 receptor on endothelial cells and promotes endothelial stabilization (144). By contrast, ANGPT2, released by activated endothelial cells, causes vascular permeability. Human basophils constitutively express ANGPT1 and ANGPT2 mRNAs (105). *In vitro* basophil activation causes the release of ANGPT1. Hepatocyte growth factor (HGF) is one of the most powerful angiogenic factors (145) and human basophils are a major source of HGF (106). Recently, it has been demonstrated that mouse lung-resident basophils express a specific gene signature including *Hgf* (44).

The cysteinyl leukotrienes (cys-LTs) are lipid mediators initially characterized for their proinflammatory activities (146). The cys-LTs include leukotriene C_4_ (LTC_4_), LTD_4_, and LTE_4_. LTC_4_ is *de novo* synthesized by several immune cells (146, 147) and is the major lipid mediator produced by activated human basophils (83, 115). LTC_4_ is converted by the extracellular enzymes, γ-glutamyl transpeptidases to LTD_4_ and to LTE_4_ by the membrane-bound dipeptidases (146). Cys-LTs activate three distinct receptors (CysLTRs) CysLT_1_R, CysLT_2_R, and CysLT_3_R (148–150). Recent evidence demonstrates that LTC_4_ and LTD_4_ were equipotent in forming tubes in the Matrigel *in vitro* assay of angiogenesis (151). The proangiogenic activities of LTC_4_ and LTD_4_ were also confirmed *in vivo* and were found to be mediated by the engagement of CysLT_2_R on blood endothelial cells (BECs). CysLT_2_R deficiency and pharmacologic antagonism reduced tumor growth and the formation of lung metastases in a mouse model of Lewis lung carcinoma (151). These novel findings emphasize the importance of cys-LTs as non-canonical angiogenic factors in cancer. It is possible to speculate that LTC_4_ released by circulating basophils can activate CysLT_2_R overexpressed in tumor BECs (151), thus contributing to angiogenesis. It has been suggested that CysLT_2_R might represent a possible pharmacologic target in tumor growth and metastases formation (151).

## Formation of Extracellular DNA Traps by Basophils

Extracellular traps (ETs) are DNA structures released by activated immune cells, including neutrophils, eosinophils, mast cells, macrophages, and basophils (116, 117, 152–155). ETs released by these cells are draped with proteins from primary granules (e.g., myeloperoxidase and elastase) (156), secondary granules (e.g., lactoferrin and pentraxin 3) (156, 157), and tertiary granules (e.g., matrix metalloproteinase 9) (156). Initial studies highlighted the antibacterial activity of ETs (154, 158, 159). During the last years, there has been increasing evidence that ETs, particularly neutrophil extracellular traps (NETs), have a role in different aspects of cancer (160). For instance, it has been demonstrated that NETs can promote cancer metastasis in mouse models and in humans (161–164). Moreover, it has been found that NETs formed during lung inflammation awaken dormant cancer cells (165). Neutrophils from patients with myeloproliferative neoplasms associated with *JAK2*^V617F^ somatic mutation have an increase in NET formation and thrombosis and mice with knock-in of *JAK2*^V617F^ have an increased propensity for NET formation and thrombosis (166). Recently, we have demonstrated that anaplastic thyroid cancer cells can induce the release of mitochondrial DNA traps by viable neutrophils (167). Collectively, these studies indicate that NETs can sustain several aspects of tumor growth, the formation of metastasis, and promote cancer-associated thrombosis. Activated human and mouse basophils can form extracellular DNA traps (BETs) *in vitro* and *in vivo* (116–118). Future studies should investigate whether BETs modulate tumor growth and the formation of metastasis in preclinical models and/or in human cancer.

## Basophil-Depleted Mice to Investigate Basophil Functions *in vivo*

It seems pertinent to review the mouse models currently employed to investigate basophil functions *in vivo*. Basophil-depleted mice will certainly play a critical role in discerning the functions of this granulocyte in cancer. Indeed, several models of basophil-deficient mice have been developed and are undergoing testing for this very purpose.

Initially, studies were performed using administration of antibodies that transiently deplete basophils. These antibodies recognize either the FcεRI (MAR-1) (168) or the activating receptor CD200R3 (Ba103) (169). Although these antibodies can deplete basophils, they can also deplete/activate other cells (e.g., mast cells, DCs, monocytes) expressing FcεRI (169–171). Furthermore, Ba103 is FcR-dependent and might activate myeloid cells and NK cells (168). Studies using these depleting antibodies have led to the controversial conclusion that basophils have a role as antigen-presenting cells (APCs) during Th2 polarization (95, 172, 173). Several new mouse strains with constitutive or inducible depletion of basophils have recently been generated (119). The Bas-TRECK and the *Mcpt8*^DTR^ are two diphtheria toxins (DT)-inducible basophil depletion mice models (125, 174). The latter models are characterized by a transient depletion of more than 90% of basophils. The *Mcpt8*^DTR^ mice express the human diphtheria toxin (DT) receptor (DTR), which makes it possible to induce a transient (~ 5 days) depletion of basophils after intraperitoneal treatment with DT (125). The *Mcpt8* gene is specifically expressed by basophils (175, 176) and encodes mouse mast cell protease 8 (mMCP-8), a granzyme B-like protease stored in the secretory granules of basophils (175). Although the expression of Mcpt8 is specific to basophils among mature cells, it is still transiently expressed at the progenitor stage to a sufficient level to allow their depletion by a high dose of DT in the *Mcpt8*^DTR^ mice (177). Injection of DT in Bas-TRECK mice also causes efficient (≥90%) depletion of basophils (174). In this model, the human DTR was inserted under control of the 3' proximal enhancer in the *IL4* locus.

Basoph8 (*Mcpt8*^IRES−YP−Cre^) (178), *Mcpt8*-Cre (179) and *P1-Runx1* (180) are three different mouse models showing constitutive depletion (~90%) of basophils. The *Mcpt8*-Cre model was developed by engineering a bacterial artificial chromosome transgenic mouse that expresses the Cre recombinase under control of the regulatory elements of *Mcpt8* (179). *Mcpt8*-Cre mice are constitutively deficient for basophils; therefore, this model is suitable for experiments that need long-term ablation of these cells. In the Basoph8 (*Mcpt8*^IRES−YP−Cre^) mice an IRES-YFP-Cre cassette was inserted before the start codon of the *Mcpt8* gene (178). The dysruption of the distal (P1) promoter of the transcription factor Runx1 resulted in >90% depletion of basophils indicating that Runx1 plays a critical role in the development of mouse basophils (180). *Runx1*^P1N/P1N^ mice have markedly reduced numbers of basophils in bone marrow, spleen and peripheral blood (180). Recently, a new mouse model (*Mcpt8*^ì*Cre*/+^*Il4*^fl/fl^) was established by crossing two mouse stains, *Mcpt8*^iCre/+^ and *Il4*^fl/fl^ mice (74). These mice are selectively deficient for IL-4 only in basophils and are thus suitable to assess the role of basophil-derived IL-4 in different pathophysiological conditions, including cancer. Several excellent reviews have analyzed in details the different mouse models to investigate basophil functions *in vivo* (75, 119, 181, 182).

It is important to emphasize that previous studies using antibody-depleted basophils (114) and genetically engineered models (62, 91) provided contrasting results on the role of basophils in cancer. Moreover, it should be pointed out that even new mouse mutants have some hematological abnormalities (177). Therefore, results obtained with basophil-deficient mouse models should be interpreted with caution.

## Peripheral Blood Basophils and Human Cancer

It has been well-known for some time that basophilia can occur during the advanced phase of chronic myeloid leukemia (CML) (183). The transcription factor IKAROS is markedly reduced in bone marrow from CML patients (184). Overexpression of the dominant-negative isoform of IKAROS in CD34^+^ cells from CML patients resulted in inhibition of IKAROS activity and increased differentiation into basophils (184). Basophils from CML patients express HGF, which promotes CML cell expansion in an autocrine fashion (106). In a mouse model of CML it has been shown that basophil-like leukemia cells promote CML development by producing the chemokine CCL3 (185). In this model basophil-derived CCL3 negatively regulates the proliferation of normal hematopoietic stem/progenitor cells and promotes the expansion of leukemia cells (186). There is also evidence that basophilia is an independent risk factor for evolution of myelodysplastic syndrome to acute myeloid leukemia (187, 188).

Peripheral blood basophils have also been associated with certain solid tumors (189). Basopenia appears to be associated with poor prognosis of colorectal cancer (190, 191), whereas circulating basophils have no predictive role in breast cancer (192), ovarian cancer (54) and oral squamous cell carcinoma (193). Of note, high relative circulating basophils positively associated with improved outcome in melanoma patients undergoing immunotherapy with nivolumab plus ipilimumab (194). On the other hand, baseline basophil count may predict recurrence in patients with high-grade bladder cancer receiving bacillus Calmette-Guérin (BCG) following resection (195). Finally, in a mouse model of breast cancer, a low percentage of circulating basophils correlated with an increased number of pulmonary metastases, suggesting a protective role of basophils in this model (196).

### Basophils and Polycythemia Vera

Polycythemia vera (PV) is a myeloproliferative neoplasm characterized by clonal stem cell proliferation of erythroid, megakaryocytic, and myeloid cell lines (197, 198). An activating Janus kinase 2 (JAK2) mutation (JAK2^V617F^ or exon 12 mutation), leading to an overactive JAK-STAT signaling pathway is found in more than 90% of PV patients (199, 200). Pruritus is a common symptom in PV patients (198, 201) and basophil-derived mediators have been implicated in this disorder (202). Absolute basophil counts have been found increased in JAK2^V617F^-positive PV patients compared to control subjects (203). The expression of CD63, a surface marker of basophil activation, is increased in PV patients with pruritus compared to controls. Finally, PV basophils are hyperresponsive to IL-3 compared to basophils from normal donors. Collectively, these findings indicate that JAK2^V617F^ mutation is associated with hyperreactivity of PV basophils. The latter observation is likely responsible for pruritus in PV patients. Given the role of basophils as major source of Th2 cytokines (e.g., IL-4), we cannot exclude the possibility that the hyperresponsiveness of these cells might play a role in the possible evolution of PV patients.

### Basophils and Ovarian Cancer

In a recent study, Bax and co-workers examined the role of basophils in ovarian cancer patients (204). They found that higher percentage of circulating basophils from ovarian cancer patients was positively associated with improved overall survival. Furthermore, by protein and gene expression analyses they detected resting (CCR3, CD123, FcεRI) and activated basophils (CD63, CD203c) in ovarian tumors. Whereas, gene expression for tumor-resident basophils was not associated with patient survival outcomes, gene signatures for activated basophils were positively associated with improved progression-free and overall survival. This study suggests that activated basophils, either in circulation or in tumor, are associated with a survival benefit in ovarian cancer patients.

## Basophils and Lung Cancer

It has been well-known for some time that murine (62, 73, 124, 125) and human (127–131) basophils have a propensity to migrate into the site of inflammation, including the lung. Whether this influx contributes to the supply of tissue resident basophils that promote M2 polarization of lung macrophages (44) remains to be determined. Nonetheless, the evidence that lung-resident basophils acquire the expression of several cytokines due to the exposure to lung-specific signals (e.g., IL-33, GM-CSF), emphasizes the plasticity of these cells. Thus, basophils migrating into tissue may take on completely new roles, based on the cytokine environment they encounter. The observation that the pulmonary microenvironment may condition the transcriptional and functional development of immune cells has recently been extended to the oncological context. Single-cell transcriptomics of human and mouse lung cancers revealed that blood and tumor neutrophils and monocytes strongly differed in their gene expression (205). Interestingly, basophils were present in mouse lung tumors. Lavin and collaborators compared the simultaneous single-cell analysis of the immune compartments in early (stage I) lung adenocarcinoma, non-involved lung tissue (nLung), and peripheral blood of each patient (50). Basophils were present in both solid tumor site and nLung. A percentage of basophils in the tumor were PD-L1^+^. This study demonstrates that, as early as in stage I disease, basophils are present in the immune landscape of nLung adenocarcinoma.

In a related example of how the TME can influence basophil function, Schroeder and collaborators demonstrated that highly purified human basophils release histamine and produce IL-4 and IL-13 when co-cultured with the lung carcinoma cell line, A549 (30). Remarkably, these responses required that basophils express IgE, yet occurred independently of allergen, and were suppressed pharmacologically by inhibitors of FcεRI signaling. It was subsequently determined that the IgE-binding lectin, galectin-3, expressed on the A549 cells, was responsible for basophil activation (206). In support of these findings, basophils co-cultured with microspheres coated with galectin-3 also secreted IL-4 and IL-13. Galectin-3 is implicated as a biomarker and/or factor contributing to the pathogenesis of a wide range of conditions, including cancer, cardiovascular disease, autoimmunity, wound healing, and chronic inflammation in general (207). Overall, these findings illustrate a novel mechanism by which galectin-3 expressed by human lung carcinoma cells can activate basophils (and likely other cell types) to release several immunoregulatory cytokines and proinflammatory mediators. Additional studies are required to elucidate the exact role of galectin-3 in activating basophils, and how the mediators and cytokines released by these cells contribute to human and experimental lung cancer.

## Basophils and Melanoma

The role of basophils has been evaluated in a mouse model of melanoma in which Treg depletion was induced (114). Treg depletion in Foxp3^DTR^ mice was associated with tumor infiltration of basophils and CD8^+^ T cells leading to rejection of melanoma. Basophils promoted CD8^+^ lymphocyte infiltration into the tumor through the production of CCL3 and CCL4. Depletion of basophils, through administration of MAR1 (i.e., anti-FcεRI), in Foxp3^DTR^ melanoma-bearing mice prevented the rejection of melanoma, suggesting a pivotal role of basophils in this model. However, as previously mentioned, MAR1 can also deplete/activate other immune cells (e.g., mast cells, DCs, monocytes) expressing FcεRI (170, 171). Thus, the possible role of basophils in melanoma will need to be confirmed using the newer genetically engineered basophil-deficient mouse models.

We recently explored the anti-tumor activity of IL-33, a cytokine known to induce tumoricidal functions in eosinophils (208, 209) on bone marrow-derived murine basophils. Incubation of basophils with IL-33 upregulated granzyme B mRNA and the surface expression of CD63 (80), indicating phenotypic and functional activation. When IL-33-activated basophils were co-cultured with metastatic B16-F10 melanoma cells, tumor cell-growth was substantially inhibited, as compared to melanoma cells co-cultured with resting basophils. These preliminary findings suggest that, under appropriate stimulation, basophils can acquire tumoricidal properties *in vitro*. Whether similar activity occurs *in vivo* remains to be determined, but it is an area of ongoing investigation.

## Basophils and Pancreatic Cancer

**Figure 2:**
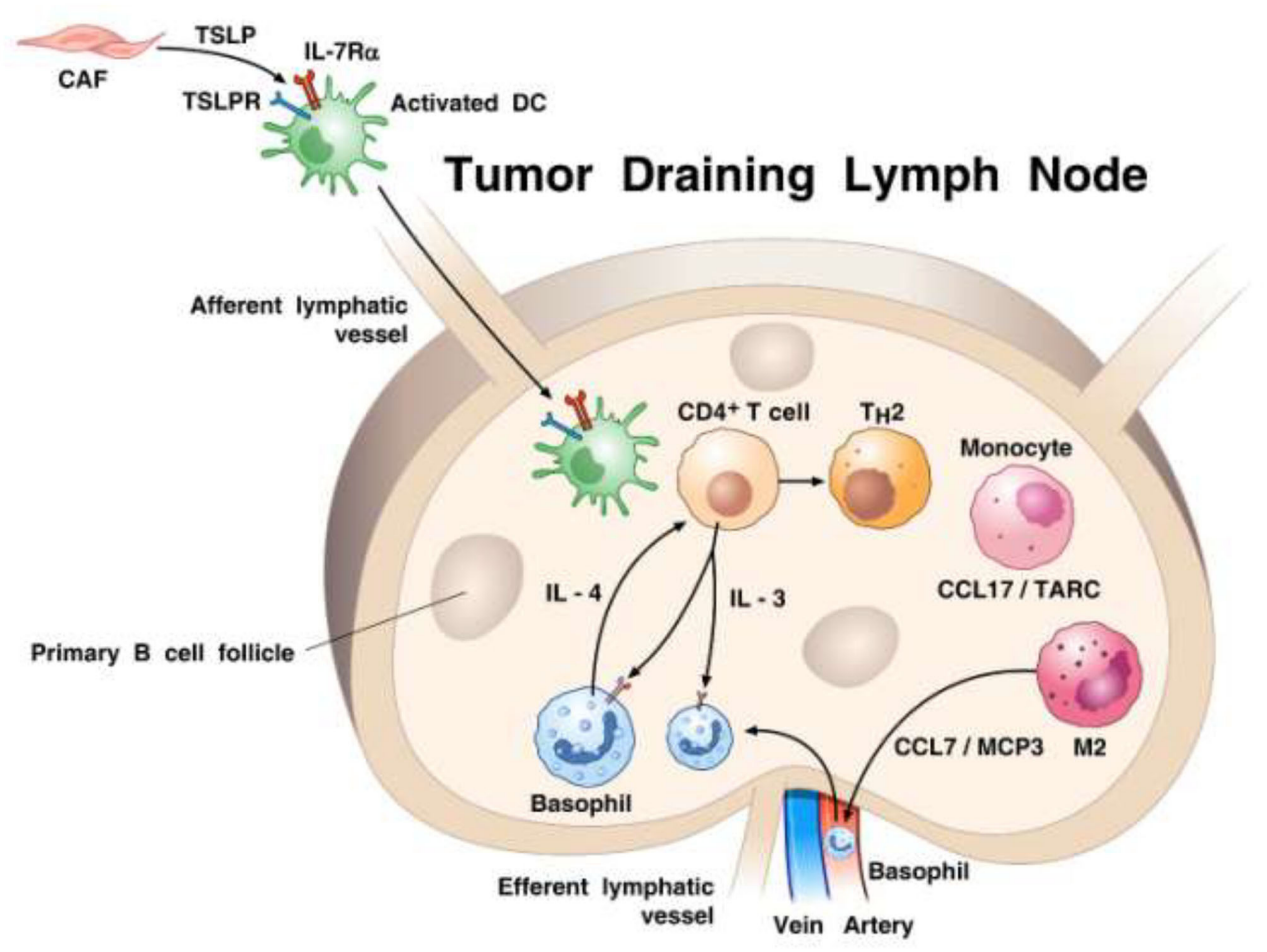
Proposed model of how basophils are recruited and activated in tumor draining lymph nodes (TDLNs) in the context of pancreatic cancer. It has been previously demonstrated (210) that cancer-associated fibroblasts (CAFs) can produce TSLP that engages TSLP receptor on dendritic cells (DCs). TSLP-conditioned DCs migrate into TDLNs were they prime CD4^+^ T cells for early IL-3 production. Monocytes, which are driven to differentiate toward a M2-type by activated CAFs, release the basophil chemoattractant CCL7/MCP3 (52). Basophils are recruited from afferent arterial blood into lymph nodes and are activated by IL-3 to express IL-4. Basophils are a major source of IL-4 contributing to both Th2 and M2 polarization. The percentage of basophils in TDLNs is an independent negative prognostic factor of survival after surgery of pancreatic cancer patients (91).

In the mid 1990s, Ann M. Dvorak showed ultrastructural features of piecemeal degranulation of human basophils in the stroma of pancreatic cancer (11). More recently, Protti and collaborators elegantly investigated the role of basophils and their mediators in experimental and human pancreatic cancer (91). In a large cohort of pancreatic ductal adenocarcinoma (PDAC) patients, they found basophils expressing *IL4* in tumor-draining lymph nodes (TDLNs) of PDAC. Importantly, the presence of basophils in TDLNs was an independent negative prognostic biomarker of patient survival after surgery. The authors also examined the possible role of basophils in an orthotopic model of pancreatic cancer using the *Mcpt8*-Cre basophil deficient (179) and WT mice. At 8 weeks after implant, tumor was found in 80% WT, but in none of basophil-deficient mice. The authors demonstrated the presence of basophils in TDLNs in this model of pancreatic cancer and provided evidence that cancer-associated fibroblasts (CAFs) released TSLP which activated DCs to produce IL-3 from CD4^+^ T cells. IL-3-activated basophils produced substantial amounts of IL-4. It was further determined that DCs and CD14^+^ monocytes produced CCL7 which was responsible for basophil migration into TDLNs. Based on these findings, schematically illustrated in Figure 2, the authors concluded that basophils can favor both Th2 and M2 polarization through the production of IL-4, thus playing a relevant pro-tumorigenic role in PDAC progression. Consistent with this latter concept of IL-4 driving M2 development, our own *in vitro* studies point to the importance of basophil-derived IL-4 (and IL-13) in promoting M2-like cells (211).

There is compelling evidence that CD4^+^ CD25^+^ Foxp3^+^ regulatory T cells (Tregs) contribute to maintain immune tolerance in the TME (212, 213) particularly in pancreatic cancer (214). A recent study has shown that Tregs can induce the expression of activation markers (CD69, CD203c, and CD13) and promote the release of several cytokines (IL-4, IL-8, IL-13) from human basophils (90). Tregs induced basophil activation through the release of IL-3. It has been suggested that Tregs might also promote tumor evasion by activating basophils to augment and sustain Th2 responses in TME by secreting IL-3 (215).

## IgE, Basophils and Skin Cancer

IgE is an ancient and the least abundant circulating immunoglobulin isotype (216). It has been suggested that IgE has evolved to provide protection against helminths (217) and environmental toxins such as venoms (218–220). Moreover, dysregulated IgE responses can cause a variety of allergic disorders (221, 222). IgE binds with very high affinity to FcεRI on mast cells and basophils and remains bound to its receptor for the life of these cells (223). It has been demonstrated that once-weekly topical application of the carcinogen 7,12-dimethylbenz [a] anthracene (DMBA) to the skin of WT mice led to the development of squamous-cell carcinomas (SCCs) after 8–15 weeks associated with high concentrations of serum IgE and infiltration of IgE-bearing basophils in skin and tumors (224). The same group of investigators extended the previous observation by demonstrating that topical application of the proinflammatory agent 12-0-tetradecanoylphorbol-13-acetate (TPA) (2x a week for 2 weeks) to the skin of WT mice increased serum IgE and IgE-bearing basophils in the skin (62). Using a two-stage inflammation driven model of epithelial carcinogenesis (DMBA and subsequent exposure to TPA) (225), they found that mice lacking IgE (*lgh7*^−/−^) were less susceptible to tumor development compared to WT mice. IgE-bearing basophils (Mcpt8^+^) accumulated inside skin tumors of WT mice. In this model, IgE-signaling was necessary for activation and histamine release from basophils. Infiltrating tissue basophils showed expression of *Cxcr2, Cxcr4*, and *Ptger2* (CRTH2, the PGD_2_ receptor). Blocking CXCR4 with a neutralizing antibody selectively reduced basophil infiltration to the inflamed skin. TSLP and IL-3, abundantly expressed in inflamed skin, increased the surface expression of CXCR4 on basophils, allowing their recruitment to the skin in response to CXCL12. Blocking TSLP and IL-3 simultaneously with neutralizing antibodies abolished basophil recruitment to the skin. The *Mcpt8*^Cre/+^ mice, which have normal mast cell numbers but strongly reduced basophils (179), were less susceptible to tumor growth. Together, these results indicate that in this inflammation-driven model of epithelial carcinogenesis, tumor promotion is mediated *via* FcεRI signaling in skin-infiltrating basophils.

## Conclusions and Outstanding Questions

For several decades, basophils were considered erroneously as primary effector cells participating solely in allergic disorders (226, 227). The concept that they might possess immunomodulatory roles became more widely appreciated when murine (5) and human basophils were shown to produce a variety of cytokines (e.g., IL-4, IL-3, and IL-13) (21, 89, 92, 93, 97, 99, 110), which at the time, were thought to be made only by Th2 cells. In addition, there is now compelling evidence that human basophils can synthesize several canonical (57, 86, 105, 106) and non-canonical angiogenic factors (151). It has long been known that human (127–131) and mouse (62, 73, 124–126) basophils have a propensity to migrate from peripheral blood into sites of inflammation. Moreover, basophils were identified in human lung (50), gastric (127, 128), pancreatic (11, 91) and ovarian cancer (204). It was recently shown, at least in mice, that basophils are present in all phases of lung development (44), and display a divergent phenotype from peripheral blood. These resident basophils can favor M2 polarization of lung macrophages, as occurs in several tumors (132, 133). Studies are urgently needed to characterize the presence and the state of activation of basophils in TME and their possible roles in early *vs*. late stages of human and experimental tumors.

Human basophils are a major source of several canonical angiogenic factors such as VEGF-A and VEGF-B (57), HGF (106), ANGPT1 (105), and CXCL8 (86, 89, 90, 228). An elegant study has recently demonstrated that LTC_4_ and LTD_4_, also produced by human basophils (83), promote tumor angiogenesis and metastasis through the engagement of CysLT_2_R on endothelial cells (151). Collectively, these findings suggest that further *in vitro* and *in vivo* investigations should evaluate the roles of canonical and non-canonical angiogenic factors produced by basophils in experimental and human tumors.

Activated human and mouse basophils release BETs (116–118). There is mounting evidence that extracellular DNA traps have multiple effects in cancer (160) favoring tumor growth (167), awakening dormant cancer cells (165), and promoting metastasis in mouse models and in humans (161, 164). Further studies should evaluate the presence of BETs in experimental and human cancers and whether basophil extracellular traps modulate tumor growth and the formation of metastasis *in vivo*.

There are contemporary and developing models/techniques that should greatly facilitate this area of investigation. For example, basophil-deficient mice are powerful models for analyzing basophil functions *in vivo*, but, in some instances, have produced erroneous findings. For example, models using antibody-depleted basophils (168, 169) can often result in the activation of other immune cells (170, 171). Indeed, such models provided highly controversial results on the role of basophils as APCs (95, 170, 172, 173, 229, 230). It is therefore not surprising that basophils may appear to play a protective (114) or a pro-tumorigenic role (62, 91) depending on the experimental model utilized. In general, mouse models with constitutive or inducible basophils depletion should be preferred, but need to take into consideration that even new mouse mutants can have hematologic abnormalities (177) and/or show incomplete removal of basophils. Studies attempting to evaluate basophil functions in a complex and heterogeneous disorder, such as cancer should be performed using multiple genetically engineered models of basophil deficiency.

In conclusion, the last years have witnessed exceptional progress in our understanding of basophil biology. Recent studies have demonstrated that basophils are present in the immune landscape of human (50, 91, 204) and experimental (62, 91) tumors, play a role in lung development and M2 macrophage polarization (44), and participate in canonical (57, 105, 106, 145) and non-canonical angiogenesis (151), and release BETs (117, 118). Further investigations are required before we unravel the mysterious role of basophils in experimental cancer and, more importantly, in humans. The elucidation of basophil role in tumor immunity will require studies of increasing complexity beyond those assessing their microlocalization. High dimensional analysis, particularly single-cell RNA-seq of immune landscape of human and experimental tumors will be of paramount importance in characterizing basophil role in different human and experimental cancers.

## Author Contributions

All authors contributed to reviewing the current literature and writing of the manuscript.

## Conflict of Interest

The authors declare that the research was conducted in the absence of any commercial or financial relationships that could be construed as a potential conflict of interest.
